# Distribution and influencing factors on residual pockets of the teeth in patients with periodontitis following non-surgical periodontal treatment: a retrospective observational study

**DOI:** 10.1186/s12903-023-03248-9

**Published:** 2023-10-09

**Authors:** Xue Yang, Peicheng Liu, Xiaomiao Fan, Shiwen Yu, Chen Chen, Yaping Pan, Li Lin, Xiaolin Tang, Chen Li

**Affiliations:** 1 Department of Periodontics, School and Hospital of Stomatology, China Medical University, No.117 Nanjing North Street, Heping District, Shenyang, Liaoning 110002 China; 2 School of Stomatology, Dalian Medical University, Dalian, Liaoning China; 3 Department of Stomatology, Tongji Medical College, The Central Hospital of Wuhan, Huazhong University of Science and Technology, Wuhan, Hubei China

**Keywords:** Periodontitis, Non-surgical periodontal treatment, Influencing factors, Prognosis

## Abstract

**Background:**

Periodontitis is a chronic and multi-factorial infectious disease. A notable difference exists in the prognosis of patients with severe periodontitis after non-surgical periodontal treatment. Thus, a retrospective study was conducted to identify common and specific factors that impact the prognosis of patients with periodontitis stage III-IV following non-surgical periodontal treatment at different tooth sites.

**Methods:**

A total of 977 teeth were included in the study, comprising 266 patients diagnosed with periodontitis stage III-IV. This sample included 330 anterior teeth, 362 maxillary posterior teeth, and 285 mandibular posterior teeth. Following treatment, the teeth were categorized into two groups based on residual pocket depth [probing depth (PD) ≥ 5 mm] at 3 months post-treatment. The prognosis of periodontitis stage III-IV was assessed through multivariate analysis employing logistic regression to determine the association of various risk factors.

**Results:**

The PD values of each site and the deepest PD values of each tooth significantly decreased at 3 months post-treatment. Residual pockets were predominantly found in the mesio/disto-buccal and mesio/disto-lingual regions. Multivariate analysis revealed that gender, PD, sulcus bleeding index (SBI) and plaque index (PLI) at baseline, and crown-root ratio in anterior teeth had a significant influence on periodontitis stage III-IV (*P* < 0.05). Smoking, PD, PLI and furcation involvement (FI) at baseline, PLI at 3 months post-treatment, grades of periodontitis, and crown-root ratio were prediction factors for maxillary posterior teeth. Factors such as PD, PLI and FI at baseline, PLI at 3 months post-treatment, and crown-root were significant in mandibular posterior teeth.

**Conclusions:**

The outcome of non-surgical treatment varies depending on the tooth positions for patients with periodontitis stage III-IV. Dentists must accurately identify the affected teeth that have periodontal pockets of more than 5 mm, taking into consideration the positions of the affected teeth, as well as various local and systemic factors. This comprehensive assessment will enable dentists to develop a customized and effective treatment plan.

**Supplementary Information:**

The online version contains supplementary material available at 10.1186/s12903-023-03248-9.

## Introduction

It is now well established that periodontal disease is a bacterial infection disease predominantly involved with dental plaque. Periodontitis is characterized by various clinical markers such as periodontal pocketing, gingival bleeding, clinical attachment loss, and alveolar bone loss [1], which if left untreated may eventually lead to tooth mobility or loss [2]. Previous studies have identified periodontitis as one of the most prevalent oral diseases globally, with approximately 10% of the population suffering from severe periodontitis [3]. Recent research conducted by Eke has found that between 2009 and 2012, 46% of adults over 30 years old in the United States were affected by periodontitis, with 8.9% reporting severe periodontitis [4]. Another investigation, the Fourth National Oral Health Survey of China conducted in 2017 revealed that only 9.1% of the 35-to-44-year-old population were periodontal health [5].

In addition to dental plaque, various factors play a crucial role in the onset and progression of periodontitis including local, systemic, demographic, and behavioral host conditions [6]. Local factors, such as tooth mobility, root bifurcation, occlusal trauma, and crown-to-root ratio, can cause periodontal inflammation and increase the risk of tooth loss [7–10]. Moreover, infected pulp, due to root canal lateral branches and dentin tubules, can spread and lead to periodontal inflammation [11]. Additionally, systemic conditions, such as diabetes, atherosclerotic cardiovascular disease, oral cancer, chronic obstructive pulmonary disease, and Alzheimer’s disease are also confirmed to be associated with periodontitis [12–15]. Large epidemiologic surveys revealed that men, aging, low education levels, and poverty were at high risk of periodontitis [16–19]. Research has shown that men, older individuals, those with low education levels, and individuals living in poverty are at a higher risk of periodontitis. Additionally, smoking is a significant risk factor for periodontitis, confirmed in both horizontal and longitudinal studies [20, 21]. Smokers experience a higher prevalence and severity of periodontitis than non-smokers, and their oral hygiene is usually poor [22, 23]. As a result, the prognosis for periodontal treatment in smoking patients is generally poor. Therefore, these factors should be carefully considered in the diagnosis and treatment of periodontitis.

Periodontal treatment is primarily aimed at eliminating bacterial agents and reducing or halting inflammation caused by pathogenic factors [24]. The two main methods of treatment are non-surgical periodontal treatment and surgical periodontal treatment. Non-surgical periodontal treatment is the most conventional approach to treating periodontitis and is the first step toward reducing bacterial infection and inflammation of the supporting tissues [24]. In case where patients with severe periodontitis still have residual pockets of 5 mm or more after non-surgical periodontal treatment, endoscopically-assisted subgingival scaling, laser therapy, or even periodontal surgery may be necessary. These methods act to eliminate infectious substances and smooth the root surface by direct visualization to maintain long-term efficacy [25]. However, these treatments require more visits, a longer course of treatment, and impose a greater financial burden on the patients. Hence, it is important to evaluate the prognosis of non-surgical periodontal treatment while making treatment plans and discussing them with patients.

Currently, numerous studies have been conducted to identify the prediction factors of periodontitis following non-surgical periodontal treatment using univariate analysis [26–28]. However, it is important to note that these factors may be interrelated, necessitating a comprehensive analysis. Additionally, different tooth sites have their own unique characteristics, leading to potentially differing prediction factors. Thus, it is crucial to refine the prediction factors for distinct dental positions through a comprehensive analysis. In this study, we categorized teeth into three regions: the anterior region, maxillary posterior region, and mandibular posterior region. Subsequently, the teeth within each region were further divided into two groups based on the depths of residual periodontal pockets after non-surgical treatment; one group with probing depth (PD) ≥ 5 mm and another with PD < 5 mm. By employing univariate analysis and multivariate analysis, we aimed to identify common and specific factors influencing the prognosis of periodontitis stage III-IV following non-surgical periodontal treatment. These findings will serve as a foundational understanding of the prognosis of teeth with periodontitis stage III-IV in various regions.

## Materials and methods

### Participant selection

A retrospective study was conducted based on records of patients with periodontitis stage III-IV who underwent non-surgical periodontal treatment and had a minimum of 5 mm of periodontal pocket depth. The patients were selected from the Department of Periodontics at the School of Stomatology, China Medical University, Shenyang, China, between September 2015 and March 2018[29]. Prior to registration, all patients had given written informed consent, and the study protocol was approved by the ethics committee of China Medical University. The study was also registered on the World Health Organization International Clinical Trials Registry Platform on 25/05/2019 (ChiCTR1900023381) and conducted in accordance with the principles of the Declaration of Helsinki. This research was carried out in compliance with the STROBE guidelines (shown in Supplementary Table 1).

### Inclusion/Exclusion criteria

All the patients included in the study met the following criteria: (1) adults (aged > 18 years); (2) systemically healthy; and (3) all the patients with periodontitis stage III-IV [29] had received non-surgical periodontal treatment, followed by supportive periodontal treatment (SPT) for a minimum of 3 months [30]. On the other hand, certain criteria were used to exclude potential participants from the study. These included: (1) pregnancy or breastfeeding; (2) systemic diseases such as hypertension or osteoporosis, or the use of medications that could affect bone metabolism; (3) the recent use of antibiotics within the past 3 months; (4) anatomically abnormal teeth such as those with invaginated lingual fossa or abnormal root morphology; (5) any prior periodontal therapy or surgery within the last 6 months; (6) traumatic teeth before/during non-surgical periodontal treatment; (7) ectopic teeth that could hind oral hygiene maintenance; (8) ill-fitting prosthesis or overhangs; and finally, (9) teeth with orthodontic treatment during non-surgical periodontal treatment [31, 32].

### Study outcomes

The primary outcome of this study was to assess the prediction factors affecting the efficacy of non-surgical periodontal treatment of patients with periodontitis stage III and stage IV. The study compared changes in various clinical parameters (PD, CAL, SBI, TM, PLI, FI) between baseline and 3 months re-evaluation appointments. Then the enrolled teeth were categorized into two groups based on the presence of residual pockets (PD ≥ 5 mm) at 3 months post-treatment evaluation. Furthermore, the impact of all the recorded variables on the treated teeth was evaluated. The sample size was determined using a calculation statistical software (Power Analysis and Sample Size, Version 11, Kaysville, Utah, USA) with a two-sided significance level of 0.05, power of 0.8 (1-β), and a case-control ratio of 1:1.15. To determine significant differences in outcomes at the 95% confidence level, 132 teeth (66 cases and 66 controls) were considered necessary.

### Demographic and clinical assessment

The demographic information was obtained from all patients, and a clinical periodontal examination was performed. The detailed demographic information included age, gender, history of cigarette smoking, and systemic history. Based on smoking status, patients were categorized as non-smokers or smokers. Non-smokers were defined as those who had never smoked or had quit smoking 5 years ago or more before diagnosis, while smokers were defined as those who continued smoking or had quit less than 5 years ago [33, 34]. All the enrolled teeth were divided into three categories: anterior teeth, maxillary posterior teeth, and mandibular posterior teeth. These teeth were then evaluated using clinical periodontal parameters. These parameters included PD, clinical attachment loss (CAL), sulcus bleeding index (SBI), tooth mobility (TM), plaque index (PLI), the type of alveolar bone loss (horizontal bone loss or vertical bone loss), the grades of periodontitis, crown-root ratio, occlusal trauma, pulp status, crown prosthesis and furcation involvement (FI) of molars. Measurements for PD, CAL, SBI (scored from 0 to 5), and PLI (scored from 0 to 3) were recorded for six surfaces (mesio-buccal, mid-buccal, disto-buccal, mesio-lingual, mid-lingual, and disto-lingual) of each tooth using an automated disk probe (FP32, Florida Probe, Gainesville, FL). All clinical periodontal measurements were performed by three calibrated examiners. Inter-examiner reliability was ensured by comparing the measurements taken by 3 examiners on the same 35 subjects [35]. Intra-examiner reliability was assessed by comparing measurements taken by each examiner on the same 100 subjects at two different periods of the same day [35]. Intra-and inter-examiner reproducibility was evaluated by intraclass correlation coefficient (ICC)[36]. The ICC values ranged between 0.83 and 0.92 for PD, 0.77 and 0.85 for CAL. Before the clinical periodontal examination, a full mouth radiograph was taken. Other clinical parameters, such as the alveolar type of bone loss, crown-root ratio, occlusal trauma, pulp status, and FI were assessed using radiographs as previously described [37–41].

### Periodontal treatment

All the patients had received initial periodontal therapy including oral hygiene instruction, full-mouth supragingival and subgingival scaling, root planing, and SPT. During SPT, professional tooth brushing, and dental flossing and/or interdental brush use were performed by the clinician. Three months after treatment, a clinical re-evaluation was performed. A PD < 5 mm combined with a BOP of < 10% was used as the clinical endpoint of the periodontal therapy [42].

### Statistical analysis

Normality assumptions of the continuous variables were assessed using the *Kolmogorov-Smirnov* test. The enrolled teeth were divided into two groups based on the presence of residual pockets (PD ≥ 5 mm) at 3 months post-treatment. To identify the risk factors associated with the prediction of periodontitis stage III-IV, we performed univariate analysis using mean and standard deviation (independent-samples *t*-test) for qualitative variables, frequencies, and percentages (χ^2^ test) for the qualitative variables, and ranking (rank and sum test) for counting variables. Subsequently, a multivariate analysis was conducted using logistic regression to examine the influence of each factor and determine the statistically significant risk factors for the prognosis of periodontitis stage III-IV. A *P*-value of less than 0.05 was considered to indicate statistical significance. All statistical analysis was performed using the SPSS software program (version 20.0; SPSS, Chicago, IL, USA).

## Results

### Study population

A total of 266 patients and 977 teeth were enrolled in this study. It included 330 anterior teeth from 73 patients (27 males and 46 females), 362 maxillary posterior teeth from 104 patients (36 males and 68 females), and 285 mandibular posterior teeth from 89 patients (31 males and 58 females). The characteristics of the participants at baseline were presented in Supplementary Table 2. Among them, 21 patients were smokers, 11 had diabetes, 252 patients had stage III periodontitis, and 14 had stage IV periodontitis. Additionally, 95 teeth showed vertical bone loss, 52 teeth had abnormal pulp, and there were 371 teeth with a crown ratio ≥ 1.

### Comparison of the proportion and distribution of PD ≥ 5 mm sites between baseline and 3 months post-treatment

The PD values of each site at baseline and 3 months post-treatment were summarized and presented in Table 1. It was evident that, in comparison to baseline, the initially diseased sites experienced a reduction in PD at 3 months post-treatment. Specifically, in anterior teeth and maxillary posterior teeth, the main concentration of PD values decreased from 4 ~ 6 mm at baseline to 2 ~ 4 mm post-treatment. Similarly, in mandibular posterior teeth, the values decreased from 3 ~ 6 mm to 2 ~ 4 mm. Additionally, at baseline, there were 1009 (50.66%) sites of anterior teeth, 1126 (51.85%) sites of maxillary posterior teeth, and 895 (52.11%) sites of mandibular posterior teeth with PD ≥ 5 mm. However, at 3 months post-treatment, these numbers decreased to 173 (8.74%) sites for anterior teeth, 409 (18.83%) sites for maxillary posterior teeth, and 281 (16.42%) sites for mandibular posterior teeth.

**Table 1:** Comparison of the PD values in each site between baseline and 3 months post-treatment

PD (mm)	Anterior teeth		Maxillary posterior teeth		Mandibular posterior teeth	
	baseline n (%)	3 months post-treatment n (%)	baseline n (%)	3 months post-treatment n (%)	baseline n (%)	3 months post-treatment n (%)
1	73 (3.69)	278 (14.04)	39 (1.80)	125 (5.76)	20 (1.17)	109 (6.37)
2	270 (13.64)	679 (34.29)	238 (10.96)	542 (24.95)	157 (9.18)	404 (23.63)
3	296 (14.95)	595 (30.05)	347 (15.98)	704 (32.41)	315 (18.42)	585 (34.21)
4	336 (16.97)	255 (12.88)	422 (19.06)	392 (18.05)	323 (18.89)	331 (19.36)
5	340 (17.17)	102 (5.15)	414 (19.06)	237 (10.91)	333 (19.47)	162 (9.47)
6	379 (19.14)	53 (2.68)	415 (19.11)	98 (4.51)	345 (20.18)	65 (3.80)
7	175 (8.84)	14 (0.71)	178 (8.20)	44 (2.03)	124 (7.25)	24 (1.40)
8	64 (3.23)	2 (0.10)	61 (2.81)	15 (0.69)	53 (3.10)	17 (0.99)
9	24 (1.21)	2 (0.10)	31 (1.43)	8 (0.37)	29 (1.70)	5 (0.29)
10	15 (0.76)	0 (0)	19 (0.87)	4 (0.18)	7 (0.41)	3 (0.18)
> 10	8 (0.40)	0 (0)	8 (0.37)	3 (0.14)	4 (0.23)	5 (0.29)
Total	1980 (100)	1980 (100)	2172 (100)	2172 (100)	1710 (100)	1710 (100)

We also compared the deepest PD values in each site before and after non-surgical periodontal treatment. The detailed data are presented in Table 2. Based on the results, we observed that the deepest PD values were predominantly in the range of 6 ~ 8 mm, 6 ~ 7 mm, and 6 ~ 8 mm in anterior teeth, maxillary posterior teeth, and mandibular posterior teeth respectively at baseline. However, after 3 months treatment, these values decreased to 3 ~ 5 mm, 3 ~ 6 mm, and 3 ~ 5 mm, respectively. It can be concluded that the deepest PD values of all the anterior teeth, maxillary posterior teeth, and mandibular posterior teeth were more than 5 mm at baseline. However, after 3 months of treatment, the deepest values of 106 (32.13%) of anterior teeth, 194 (53.58%) of maxillary posterior teeth, and 134 (47.01%) of mandibular posterior teeth were still more than 5 mm.

**Table 2:** Comparison of the deepest PD values between baseline and 3 months post-treatment

Deep-est PD (mm)	Anterior teeth		Maxillary posterior teeth		Mandibular posterior teeth	
	baseline n (%)	3 months post-treatment n (%)	baseline n (%)	3 months post-treatment n (%)	baseline n (%)	3 months post-treatment n (%)
1	0 (0)	0 (0)	0 (0)	1 (0.28)	0 (0)	1 (0.35)
2	0 (0)	12 (3.64)	0 (0)	6 (1.66)	0 (0)	5 (1.75)
3	0 (0)	125 (37.88)	0 (0)	64 (17.68)	0 (0)	62(21.75)
4	0 (0)	87 (26.36)	0 (0)	97 (26.80)	0 (0)	83 (29.12)
5	0 (0)	59 (17.88)	0 (0)	92 (25.41)	0 (0)	66 (23.16)
6	159 (48.18)	34 (10.30)	199 (54.97)	56 (15.47)	148(51.93)	35 (12.28)
7	98 (29.70)	9 (2.73)	93 (25.69)	25 (6.91)	71 (24.91)	13 (4.56)
8	45 (13.64)	2 (0.61)	33 (9.12)	10 (2.76)	35 (12.28)	11 (3.86)
9	16 (4.85)	2 (0.61)	19 (5.25)	5 (1.38)	23 (8.07)	4 (1.40)
10	7 (2.12)	0 (0)	12 (3.31)	4 (1.10)	5 (1.75)	3 (1.05)
> 10	5 (1.52)	0 (0)	6 (1.66)	2 (0.55)	3 (1.05)	2 (0.70)
Total	330 (100%)	330 (100%)	362 (100%)	362 (100%)	285 (100%)	285 (100%)

**Fig. 1:**
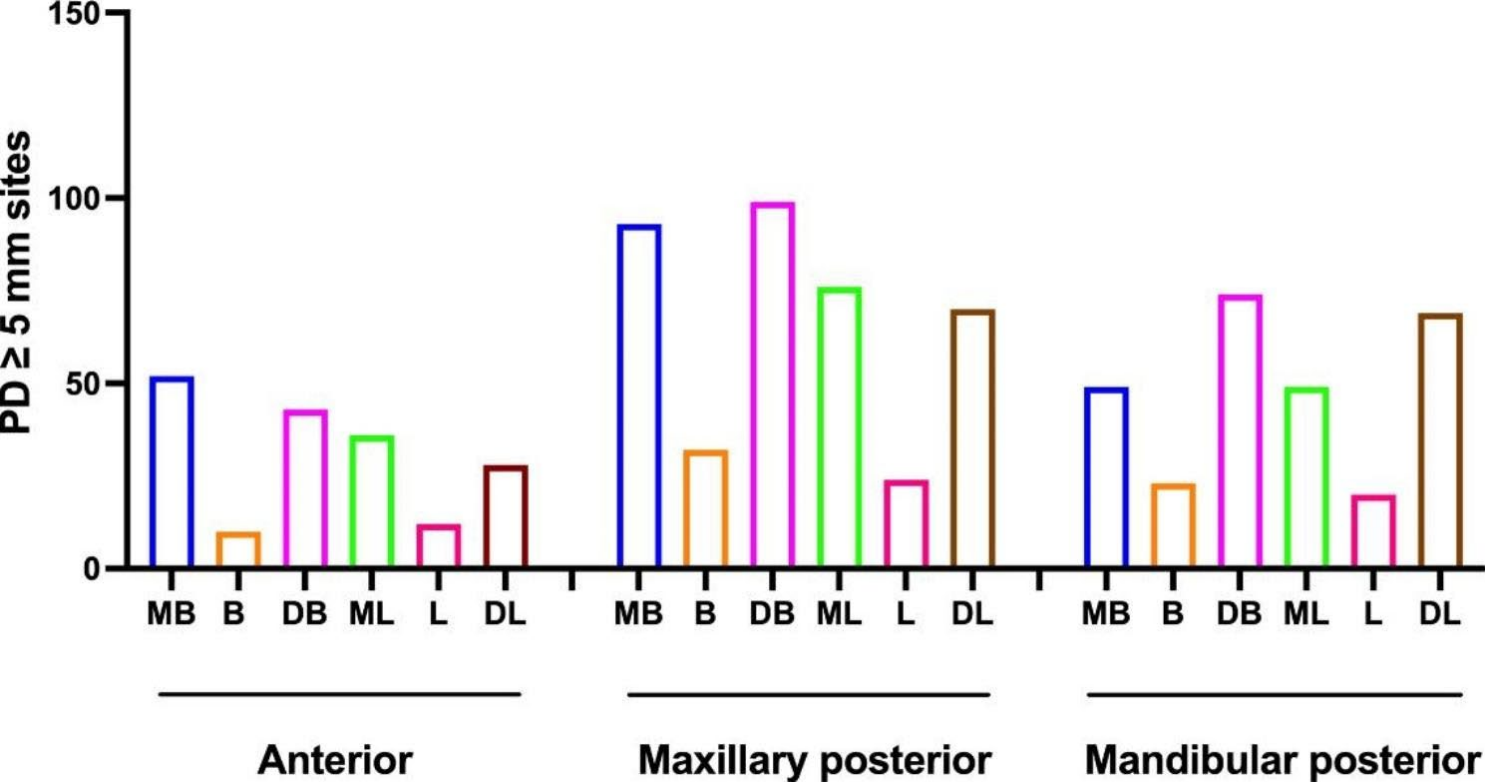
Comparison of the distribution of PD ≥ 5 mm sites in anterior, maxillary posterior, and mandibular posterior teeth. MB, mesio-bucca; B, mid-buccal; DB, disto-buccal; ML, mesio-lingual; L, mid-lingual; DL, disto-lingual

Then we generalized the distribution of PD ≥ 5 mm sites in Fig. 1. Among anterior teeth, PD ≥ 5 mm sites were primarily concentrated at the mesio-buccal (238) and disto-buccal (238) at baseline, and at the mesio-buccal (52) at 3 months post-treatment. In maxillary posterior teeth, PD ≥ 5 mm sites were mainly concentrated at the disto-buccal (265) and mesio-buccal (257) aspects at baseline, and at the disto-buccal (99) and mesio-buccal (93) positions at 3 months post-treatment. Among mandibular posterior teeth, PD ≥ 5 mm sites were predominantly found at the disto-buccal (203) and disto-lingual (197) aspects at baseline, and at the disto-buccal (74) and disto-lingual (69) at 3 months post-treatment. It is worth noting that PD ≥ 5 mm sites were observed at the mid-buccal and mid-lingual regions in all three areas.

### **Univariate analysis of the risk factors affecting the prognosis of the teeth with PD** ≥ **5 mm in periodontitis stage III-IV**

To identify the risk factors, all the enrolled teeth were divided into two groups based on the presence of a residual pocket at 3 months post-treatment: PD ≥ 5 mm group and PD < 5 mm group. Demographic and clinical parameters were compared between the two groups (shown in Table 3). Significant differences were observed in anterior teeth between the PD < 5 mm group and PD ≥ 5 mm group in terms of gender, smoking, values of PD, the deepest PD, SBI, and PLI at baseline, type of bone loss, and the crown-root ratio. Likewise, in maxillary posterior teeth, there were significant differences between the two groups in smoking, the value of PD, the deepest PD, CAL, TM, PLI, and FI at baseline, PLI value at 3 months post-treatment, type of bone loss, grades of periodontitis, crown-root ratio, and occlusal trauma. Finally, in mandibular posterior teeth, the significantly different factors were smoking, diabetes, the value of PD, the deepest PD, CAL, TM, PLI, and FI at baseline, PLI value at 3 months post-treatment, type of bone loss, grades of periodontitis, crown-root ratio, occlusal trauma, and pulp status.

**Table 3:** Univariate analysis of the demographic and clinical parameters between PD < 5 mm group and PD ≥ 5 mm group

PD (mm)	Anterior teeth				Maxillary posterior teeth				Mandibular posterior teeth			
	PD < 5 mm	PD ≥ 5 mm	Χχ^2^/*t*/*Z*	*P*	PD < 5 mm	PD ≥ 5 mm	χ^2^/*t*/*Z*	*P*	PD < 5 mm	PD ≥ 5 mm	χ^2^/*t*/*Z*	*P*
Number	224	106			168	194			151	134		
Age^1^	42.09 ± 13.32	43.91 ± 12.54	-1.176	0.241	43.31 ± 14.89	43.70 ± 13.96	-0.258	0.796	41.95 ± 14.95	42.71 ± 13.42	-0.622	0.059
Gender^2^			8.014	0.006^#^			2.777	0.107			0.024	0.903
Male	64	47			60	86			55	50		
Female	160	59			108	108			96	84		
Smoking^2^			11.314	0.001^#^			17.987	0.009^#^			6.040	0.017^*^
Smokers	19	23			22	62			14	26		
Non-smokers	205	83			146	132			137	108		
Diabetes^2^			0.391	0.784			0.003	1.000			7.958	0.006^#^
Yes	12	4			11	13			133	130		
No	212	102			157	181			18	4		
PD (baseline) ^1^ (Mean ± SD)	4.37 ± 0.99	4.95 ± 1.14	-4.725	0.000^#^	0.009	4.98 ± 1.06	-7.478	0.000^#^	4.44 ± 0.68	4.92 ± 0.94	-4.920	0.000^#^
Deepest PD (baseline) ^1^ (Mean ± SD)	6.72 ± 1.13	7.24 ± 1.21	-3.772	0.000^#^	6.45 ± 0.71	7.14 ± 1.46	-5.596	0.000^#^	6.60 ± 0.92	7.16 ± 1.27	-4.310	0.000^#^
CAL (baseline) ^1^ (Mean ± SD)	3.20 ± 1.53	3.40 ± 1.48	-0.087	0.930	3.05 ± 1.50	3.58 ± 1.50	-3.766	0.000^#^	3.02 ± 1.07	3.56 ± 1.39	-3.698	0.000^#^
SBI (baseline)^3^			-2.161	0.031^*^			-0.002	0.999			-0.322	0.747
0	0	0			0	0			0	0		
1	3	2			0	1			3	2		
2	35	15			27	37			26	19		
3	61	41			69	68			55	54		
4	124	43			72	88			67	58		
5	1	1			0	0			0	1		
TM (baseline) ^3^			-0.684	0.494			-4.913	0.000^#^			-3.176	0.000^#^
0	106	45			120	94			105	72		
1	69	37			37	56			32	31		
2	34	16			10	32			14	27		
3	15	8			1	12			0	4		
PLI (baseline) ^3^			-3.267	0.001^#^			-4.038	0.000^#^			-4.758	0.000^#^
0	1	2			5	0			1	0		
1	23	17			25	9			25	4		
2	73	47			58	58			50	30		
3	127	40			80	127			75	99		
PLI (3 months post-treatment) ^3^			-1.417	0.156			-5.710	0.000^#^			-4.857	0.000^#^
0	76	31			58	24			62	24		
1	129	59			85	105			73	74		
2	16	15			23	50			14	30		
3	3	1			2	15			2	6		
Bone loss^2^			14.180	0.000^#^			11.294	0.001^#^			2.758	0.252
Horizontal	220	94			145	143			137	113		
Vertical	4	12			23	51			1	21		
Grades of periodontitis ^2^			0.001	1.000			8.786	0.004^#^			1.960	0.167
II	161	76			145	143			120	97		
III	63	30			23	51			31	37		
FI (baseline)^3^							-4.987	0.000^#^			-5.270	0.000^#^
0					119	91			113	61		
1					26	41			20	27		
2					20	49			15	37		
3					2	12			3	9		
4					0	1						
Crown-root ratio^2^			12.851	0.000^#^			24.677	0.000^#^			12.171	0.001^#^
< 1	140	44			129	100			116	77		
≥ 1	84	62			39	94			35	57		
Occlusal trauma^2^			3.536				6.042	0.014^**^			5.562	0.028^*^
Yes	216	9			166	182			149	125		
No	8	97			2	12			2	9		
Pulp status^2^			1.657	0.243			3.541	0.084			5.811	0.018^*^
Abnormal	1	2			9	21			146	120		
Normal	223	104			159	173			5	14		
Crown prosthesis^2^			0.083	0.793			1.767	0.209			1.704	0.253
Yes	11	6			8	6			13	18		
No	213	100			160	178			138	116		

SD, standard deviation; PD < 5 mm group versus PD ≥ 5 mm group using ^1^ the independent *t* test, ^2^ χ^2^ test, and ^3^ rank sum test

^*^P < 0.05, ^#^*P* < 0.01

### Multivariate logistic regression analysis to analyze the influence of each factor on the prognosis of the teeth with PD ≥ 5 mm in periodontitis stage III-IV

In order to investigate the influence of each factor on the prognosis, we used multivariate logistic regression analysis to identify the factors that had a statistically significant impact on the risk (shown in Table 4). The results showed that several factors significantly affected the prognosis of anterior teeth, including gender, PD, SBI, and PLI at baseline, as well as the crown-root ratio. For maxillary posterior teeth, smoking, PD, PLI, and FI at baseline, PLI at 3 months post-treatment, grades of periodontitis, and crown-root ratio played a significant role in the prognosis. Additionally, PD, PLI, and FI at baseline, PLI at 3 months post-treatment, and crown-root ratio were found to significantly influence the prognosis of mandibular posterior teeth.

**Table 4:** Multivariate logistic regression analysis of risk factors significantly affecting the prognosis of severe chronic periodontitis

Factors	Anterior teeth			Maxillary posterior teeth			Mandibular posterior teeth		
	OR	95% CI	*P*	OR	95% CI	*P*	OR	95% CI	*P*
Gender	3.117	1.657 ~ 5.864	0.000	—	—	—	—	—	—
Smoking	—	—	—	2.433	1.230 ~ 4.814	0.011			
PD (baseline)	1.560	1.069 ~ 2.277	0.021	1.592	1.023 ~ 2.478	0.039	1.765	1.030 ~ 3.023	0.039
SBI (baseline)			0.025			—			—
2	0.260	0.049 ~ 1.378		—	—		—	—	
3	0.489	0.099 ~ 2.417		—	—		—	—	
4	0.205	0.042 ~ 1.010		—	—		—	—	
5	1.329	0.053 ~ 33.289		—	—		—	—	
PLI (baseline)			0.009			0.000			0.006
1	6.175	0.278 ~ 137.017		6.232	2.232 ~ 17.400		—	—	
2	9.021	3.291 ~ 24.727		9.021	3.291 ~ 24.727		3.217	0.879 ~ 11.771	
3	1.681	0.081 ~ 34.781		—	—		6.624	1.825 ~ 24.084	
PLI (3 months post-treatment)			—			0.000			0.019
≤ 1	—	—		2.489	1.252 ~ 4.945		2.356	1.194 ~ 4.648	
2	—	—		2.988	1.253 ~ 7.126		3.717	1.421 ~ 9.725	
3	—	—		5.534	0.980 ~ 31.238		5.982	0.850 ~ 42.078	
FI (baseline)			—			0.033			0.001
1	—	—		1.638	0.819 ~ 3.275		—	—	
2	—	—		2.555	1.271 ~ 5.136		2.105	0.965 ~ 4.592	
3	—	—		3.880	0.595 ~ 25.296		3.710	1.656 ~ 8.313	
4	—	—					9.233	1.805 ~ 47.223	
Periodontitis grades	—	—	—	2.897	1.394 ~ 6.019	0.004	—	—	—
Crown-root ratio ≥ 1	1.894	1.067 ~ 3.362	0.029	2.387	1.318 ~ 4.323	0.004	1.978	1.016 ~ 3.848	0.045

OR, odd ratio; 95% CI, 95% confidence interval

## Discussion

After non-surgical periodontal treatment, the reduction in PD can be attributed to the recession of the gingiva and gain in CAL [26]. Additionally, when PD < 5 mm, maintaining oral hygiene becomes relatively easier [43]. In this study, we examined the PD values of all the enrolled teeth. The results clearly indicated a significant decrease in both the PD values of each site and the deepest PD values of each tooth at 3 months post-treatment. These findings highlighted the evident effectiveness of conventional non-surgical periodontal treatment for individuals with periodontitis stage III-IV. Furthermore, we conducted an analysis of the distribution of residual pockets (≥ 5 mm) across different regions. The outcomes revealed that the residual pockets were predominantly found in mesio/disto-buccal and mesio/disto-lingual regions. This occurrence can be attributed to the increased difficulty in cleaning the adjacent surfaces when compared to the buccal and lingual sides. Consequently, the accumulation of plaque and dental calculus in these areas results in the formation of deep periodontal pockets.

Our results also revealed that non-surgical periodontal treatment had a more effective therapeutic effect on anterior teeth compared to posterior teeth. We identified three potential reasons to explain this. Firstly, anterior teeth had relatively simple anatomy and were positionally advantageous, making it easier for dentists to remove root plaque and calculus. Secondly, anterior teeth had fewer pits and fissures, making it easier for patients to clean the surface of these teeth during supportive therapy, thereby facilitating better maintenance of the therapeutic effect. Additionally, the thin anterior gingiva and labial bone contributed to the easy recovery of hyperplastic gingiva, leading to a reduction in PD.

From the univariate analysis and multivariate logistic regression analysis, we found that the mean PD and PLI at baseline, as well as the crown-root ratio, were common prediction factors for anterior teeth, maxillary posterior teeth, and mandibular posterior teeth. It was worth noting that the univariate analysis revealed differences in both the mean and deepest values of PD at baseline in all regions between the PD < 5 mm group and PD ≥ 5 mm group. However, the results of the multivariate logistic regression analysis showed that only the mean PD at baseline was an independent influencing factor for prognosis. These findings suggested that teeth with periodontitis stage III-IV may have a better prognosis if they only had a single site with a deep PD value and acceptable mean PD values while excluding abnormal anatomical structure and occlusion trauma. Contrary to our results, the study by Ekuni D et al.[44] identified the deepest PD, tooth mobility, and multi-rooted teeth at baseline as influencing factors for tooth loss. This discrepancy may be due to their focus on tooth preservation, whereas our study analyzed the factors affecting the presence of residual pockets 3 months after non-surgical periodontal treatment, with all teeth already preserved. Moreover, the PLI at baseline reflected the patients’ oral health status and their level of awareness regarding oral health care. Lower PLI values indicated better oral health status and a better prognosis after non-surgical periodontal treatment. In patients with periodontitis stage III-IV, the clinical crown-root ratio increased due to significant alveolar bone resorption and a decrease in periodontal supporting tissue. Consequently, the stress on the periodontal ligament gradually increased, resulting in deeper probing depth [38].

In addition to these common influencing factors, there were specific factors that influenced different regions. When it came to anterior teeth, gender and SBI at baseline served as prediction factors. The impact of gender on the prognosis of anterior teeth may be attributed to the fact that women tend to place more importance on the appearance of their anterior teeth. As a result, they are more likely to detect and promptly treat gingival redness and bleeding. SBI, on the other hand, acts as an indicator of the degree of periodontal inflammation and the activity of periodontal disease. A more severe inflammation typically leads to a worse prognosis. While SBI was easily influenced by other factors such as occlusal trauma and smoking, these factors had less of an effect on anterior teeth compared to posterior teeth. Hence, SBI at baseline only existed as a prediction factor for anterior teeth. When considering the posterior region, FI at baseline and PLI at 3 months post-treatment for both the maxillary and mandibular regions were found to be prediction factors. Prognosis in the root furcation of posterior teeth was influenced by certain anatomical factors, including the length of the root trunk, the angle of the furcation, the width at the entrance of the furcation, and the presence of root concavities, enamel projection, enamel pearls, and collateral canals. Due to its complex structure, accumulation of inflammation in the root furcation area poses difficulties in accessing the region with curettes and thoroughly removing dental plaque [45–47]. This is the primary reason for the poor prognosis of teeth with FI. During the non-surgical periodontal treatment, we conducted oral hygiene instructions for the patient whenever they visited, oral hygiene instructions were provided to patients at every visit, which included guidance on professional tooth brushing, dental flossing, and/or interdental brush use. The majority of patients improved their ability to maintain oral hygiene through this process, but there still remained some plaque retention, particularly in posterior teeth. The retention of plaque during the periodontal supporting therapy could impact the prognosis after non-surgical periodontal treatment. Therefore, the PLI at 3 months post-treatment served as the sole prediction factor for posterior teeth.

Furthermore, the prediction factors differed between maxillary posterior teeth and mandibular posterior teeth. Compared with mandibular posterior teeth, maxillary posterior teeth had two specific prediction factors, smoking and the grades of periodontitis. As for smoking, it was speculated that toxic substances in tobacco first reached the maxillary posterior teeth and altered the microenvironment in the periodontal pocket. This led to an increase in the proportion of periodontal pathogens such as *Aggregatibacter actinomycetemcomitans*, *Tannerella forsythia*, and *Porphyromonas gingivalis* increased. Additionally, smoking could disrupt the number and function of leukocytes in gingival tissue, decrease immune capacity, reduce secretion of IgG, and elevate IgE levels. These factors contributed to dysfunctional fibroblasts and difficulty in attachment to the root surface, resulting in a poor prognosis for maxillary posterior teeth in smokers. Therefore, smoking was a specific prognostic factor for maxillary posterior teeth [48, 49]. The grade of periodontitis reflected the speed of the procession of periodontitis and was influenced by age, CAL, smoking, and diabetes. The results of this study suggested that the grade of periodontitis was a specific prognostic factor for maxillary posterior teeth. Further investigation is needed to uncover the reasons behind this observation.

**Fig. 2:**
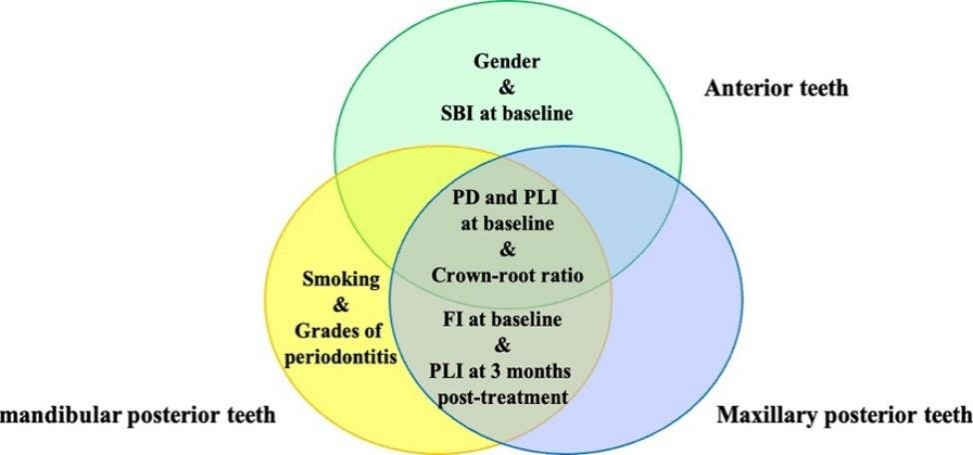
The prognostic factors of anterior teeth, maxillary posterior teeth, and mandibular posterior teeth

In this retrospective study, we analyzed the distribution of periodontal pockets and the deepest PD in different regions of the mouth (anterior, maxillary posterior, and mandibular posterior) among patients with periodontitis stage III-IV. We examined these factors before and after non-surgical periodontal treatment, and identified prognostic factors through multivariate analysis. For anterior teeth, we observed that male patients with higher mean PD, SBI, and PLI at baseline and uncoordinated crown-root ratio had a poor prognosis. In posterior teeth, aside from the crown-root ratio, higher PD and PLI at baseline, as well as higher PLI at 3 months post-treatment, and FI at baseline also indicated a poor prognosis. Regarding maxillary posterior teeth, patients who were smokers and exhibited higher grades of periodontitis were suggested to have a poor prognosis (shown in Fig. 2). Dentists should pay careful attention to patients presenting with these aforementioned factors when devising treatment plans. It is essential to fully inform patients that further treatment may be necessary for teeth with a poor prognosis despite undergoing non-surgical periodontal treatment. This study provides valuable insights into the prognostic indicators of periodontitis stage III-IV after non-surgical periodontal treatment in different regions of the mouth. Such findings are highly relevant for effective doctor-patient communication and clinical decision-making.

However, the study also have some limitations that should be acknowledged. Firstly, the retrospective design employed in this study is considered inferior to a prospective design due to the way information was gathered. Secondly, while the pulp status was recorded, the presence of caries was not included in the analysis. Furthermore, it was observed that the pulp status did not significantly impact the prognosis of periodontitis, which could be attributed to the smaller number of patients with pulp abnormalities included in the study. Therefore, future research should aim to assess a large number of patients over an extended period of time in order to provide more conclusive results.

### Electronic supplementary material

Below is the link to the electronic supplementary material.

Supplementary Material 1

Supplementary Material 2

Supplementary Material 3

## Data Availability

The datasets used and/or analyzed during the current study are available from the corresponding author on reasonable request.
