# Supplementary material for: Distribution and influencing factors on residual pockets of the teeth in patients with periodontitis following non-surgical periodontal treatment: a retrospective observational study
Source: BMC Oral Health. 2023 Oct 9;23:736. doi: 10.1186/s12903-023-03248-9 (PMC10561464; doi:10.1186/s12903-023-03248-9)
Supplement: Supplementary file 2 — Supplementary Material 2 [file 12903_2023_3248_MOESM2_ESM.docx]

Supplementary table 2. Characteristics of the participants

| Characteristics | Frequency |
| --- | --- |
| Males n (%) | 94 (35.33) |
| Age (year) | 43 ± 14 |
| Smokers n (%) | 7.89 |
| Diabetes n (%) | 4.14 |
| Baseline PD (mm) | 4.61±0.96 |
| Baseline CAL (mm) | 3.30±1.37 |
| Baseline SBI | 3.27±0.79 |
| Baseline TM | 0.66±0.85 |
| Baseline PLI | 2.44±0.72 |
| Classification of periodontitis (n) |  |
| Stage III periodontitis | 252 |
| Stage IV periodontitis | 14 |
| Teeth with occlusal trauma (n) | 42 |
| Teeth with abnormal pulp (n) | 52 |
| Teeth with prosthesis (n) | 72 |
| Bone loss (n) |  |
| Horizontal | 882 |
| Vertical | 95 |
| Crown-root ratio (n) |  |
| <1 | 606 |
| ≥1 | 371 |

Values are arithmetic mean ± SD for continuous variables.

Categorical variables are shown as numbers (n) and percentages (%). n: sample size.
