# Supplementary figures and images for: Distribution and influencing factors on residual pockets of the teeth in patients with periodontitis following non-surgical periodontal treatment: a retrospective observational study
Source: BMC Oral Health. 2023 Oct 9;23:736. doi: 10.1186/s12903-023-03248-9 (PMC10561464; doi:10.1186/s12903-023-03248-9)

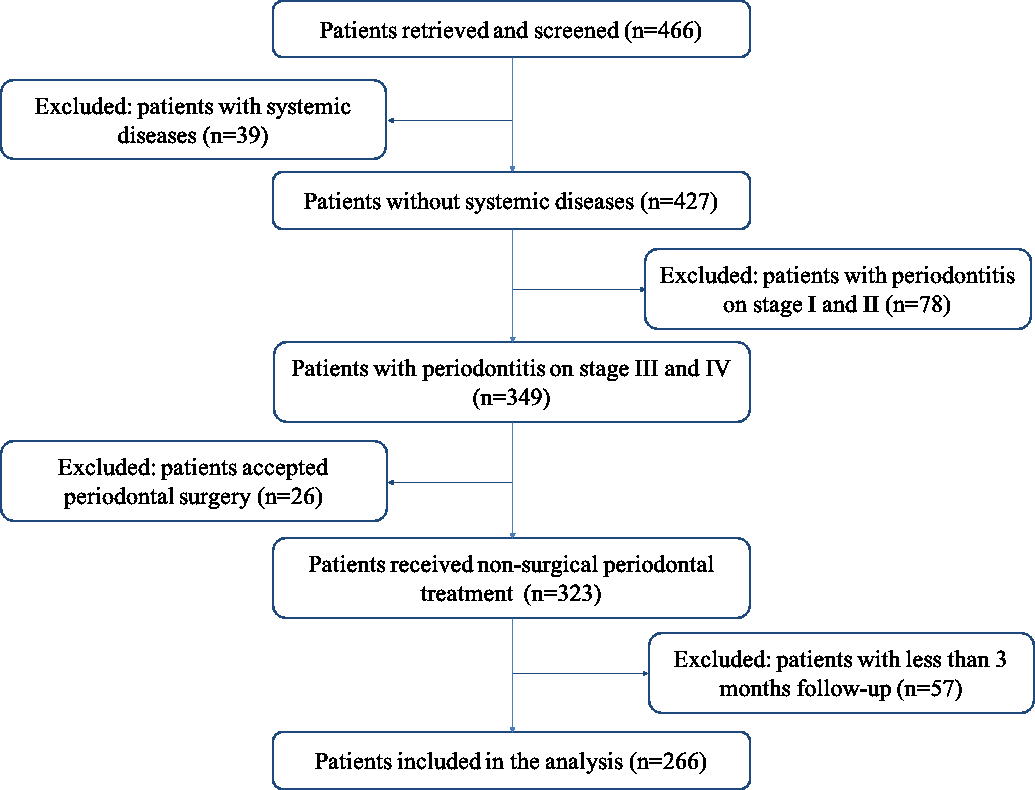


**Supplementary Figure 1**. Flowchart of the number of patients enrolled.

Supplement: Supplementary file 3 — Supplementary Material 3 [file 12903_2023_3248_MOESM3_ESM.docx]
